# Performance of the ENSEAL X1 Curved Jaw Tissue Sealer in thoracic procedures in a Japanese cohort: a case series report

**DOI:** 10.1007/s11748-023-01980-1

**Published:** 2023-10-14

**Authors:** Hiroyuki Ito, Masahiro Tsuboi, Kristy Canavan, Paula Veldhuis, Mordechai Goode Sadowsky

**Affiliations:** 1 Department of Thoracic Surgery, Kanagawa Cancer Center, Yokohama, Japan; 2 Department of Thoracic Surgery, National Cancer Center Hospital East, Kashiwa, Japan; 3 Clinical, Ethicon Inc., Cincinnati, OH USA; 4 Medical Affairs, Ethicon Inc., 4545 Creek Rd, Cincinnati, OH 45242 USA; 5 University of Cincinnati, Cincinnati, OH USA

**Keywords:** Lung resection, ENSEAL X1 Curved Jaw Tissue Sealer, Hemostasis

## Abstract

**Background:**

Advanced vessel sealing electrosurgical systems have been widely adopted for grasping, cutting, and sealing vessels. Data remain sparse with regard to its use in thoracic procedures. Thus, a prospective case series, utilizing the ENSEAL X1 Curved Jaw Tissue Sealer (X1CJ) and its companion energy source, the Generator 11 (GEN11), in thoracic procedures was performed in a Japanese cohort.

**Methods:**

Subjects were recruited at two Japanese surgical sites. The primary endpoint of this post-market study was the achievement of hemostasis (≤ Grade 3) for each thoracic vessel transection. Performance endpoints included scores for tasks completed with X1CJ (adhesiolysis, lymphatics or tissue bundles divided, tissue grasping, tissue cutting, or tissue dissection); hemostasis grading vessel transected; additional products required to achieve hemostasis for Grade 4 vessel transections. Safety was evaluated by evaluating device-related adverse events. All endpoint data were summarized.

**Results:**

Forty subjects (50.0% female) of Asian ethnicity with a mean age of 67.6 ± 11.3 years underwent a lung resection. Estimated mean blood loss was 39.5 mL. Hemostasis was achieved in 97.5% of vessel transections. Thirty-seven vessel sealings resulted in a hemostatic Grade 1 (92.5%). All surgeons reported satisfaction/neutral in terms of tissue grasping (100.0%) while most reported satisfaction/neutral with tissue cutting (95.7%). One device-related serious adverse event was reported (2.5%), a chylothorax requiring an extension of hospitalization. There was no post-operative bleeding or deaths reported during the study period.

**Conclusion:**

The X1CJ demonstrated safe and effective performance without any reports of significant intra-operative or post-operative hemorrhage in thoracic vessel sealing.

## Introduction and background

Minimally invasive thoracic surgery has become the standard for many procedures, especially the segmental and lobar resection of lung cancers [1]. However, there remain challenges in the minimally invasive environment, especially regarding handling, dissection, and division of the delicate pulmonary vessels. Endocutter stapling devices are commonly used for larger thoracic vessels, but since their size have reduced maneuverability within the confines of the thoracic cavity, they are less well-suited for division of smaller pulmonary and bronchial vessel branches [2]. Advanced vessel sealing electrosurgical systems have been widely adopted in other specialties such as metabolic/bariatric, colorectal, gynecologic, and urologic surgery. These electrosurgical systems include ultrasonic devices (including the Harmonic scalpel, Ethicon, Inc., USA), advanced bipolar feedback-controlled electrosurgical technologies (such as LigaSure™; Medtronic, USA), and combination devices (such as Thunderbeat^®^; Olympus, Japan) [3, 4]. Despite many benefits achieved through the use of the energy devices, including reduced operative time, less operative blood loss, and fewer post-operative complications, risks associated with tissue and vessel sealing with the energy devices persist including blood loss and potential thermal injury [4–6]. The performance of these devices is well established in other specialties[4, 7, 8], but there is relatively less data regarding their use in thoracic surgery.

Previously, an advanced bipolar device designed for open procedures, the ENSEAL X1 Large Jaw Tissue Sealer, has been tested successfully in a variety of open thoracic procedures [9]. The ENSEAL X1 Curved Jaw Tissue Sealer (X1CJ) is an advanced bipolar tool used to seal and cut blood vessels in minimally invasive procedures. The X1C1 jaw is designed to provide uniform compression and uses electrical impedance feedback to monitor tissue conditions and modulate energy and has separate sealing and cutting functionality.

To better understand the safety and performance of the ENSEAL X1 Curved Jaw Tissue Sealer in thoracic procedures, a case series study was performed in a Japanese cohort.

## Methods

### Study design and patient population

Forty consecutively presenting subjects were recruited at two surgical sites in Japan (Kanagawa Cancer Center; National Cancer Center Hospital East, Chiba) in this post-market, prospective, single-arm, observational case series (Clinical Trials identifier NCT05067647). Eligible patients included adults who were scheduled for any elective primary resection procedure where at least one vessel (not a main pulmonary artery or vein) was to be transected with the device per Instructions for Use (IFU). Patients and those enrolled in any other interventional clinical trials were excluded from participation.

Subjects were considered enrolled after consent was obtained, screening and baseline assessments completed, eligibility confirmed, and the use of ENSEAL X1 device had been attempted on at least one vessel transection. All subjects enrolled were followed post-operatively through discharge and again at 28 days (± 14 days) post-surgery. The study was conducted in accordance with the Helsinki Declaration, 21 CFR Part 50, and local regulations. Independent Ethics Committee approval was obtained prior to study onset and informed consent was obtained from subjects preceding study enrollment.

### Device and indication

Devices evaluated were the ENSEAL X1 Curved Jaw Tissue Sealer which is used in open and laparoscopic surgery to transect vessels up to and including 7 mm in diameter paired exclusively with the Generator G11 (GEN11) (Ethicon Inc., Cincinnati, OH).

### Endpoints

The primary performance endpoint was the achievement of hemostasis for each vessel transection. This was assessed based upon the following grading scale: Grade 1: no bleeding at transection site; Grade 2: minor bleeding at transection site with no required intervention; Grade 3: minor bleeding at transection with mild intervention required; Grade 4: significant bleeding at transection site requiring intervention. Specifically, successful hemostasis after vessel transection was defined as a Grade ≤ 3.

The secondary performance endpoints included the distribution of scores of surgeon assessment of usability on a five-point scale for various tasks completed by the C1CJ device (adhesiolysis, lymphatics or tissue bundles divided, tissue grasping, tissue cutting, or tissue dissection); distribution of hemostasis grading scale for each vessel transected; and additional hemostasis products required to achieve hemostasis for Grade 4 vessel transections. Further, a safety endpoint evaluated the occurrence of device-related adverse events (AEs). Adverse events were reported while in-patient and through the follow-up period by patient evaluation and/or patient complaint.

### Data collection

Basic baseline and demographic variables (including age, gender, height, weight, and BMI) were collected. In addition, clinical assessment of American Society of Anesthesiologists Physical Status Classification System (ASA) scores was recorded, along with the primary indication for surgery and the procedure to be performed.

Intra-operative surgical variables recorded included duration of procedure; whether vessel skeletonization occurred; potential presence of inflamed, calcified, atherosclerotic, fibrotic tissue; how and where the X1CJ was used (i.e., grasping, cutting, dissection); if adhesions were removed or divided; if minimally invasive surgery required conversion to open; estimated blood loss (EBL); if blood transfusion was needed, and if so the number of units given; and if an additional energy device aside from the study device was required. A full vessel transection summary was obtained over the course of the study which included the number, location, and size of vessels transected, whether or not hemostasis was achieved, touch-ups required, and number of additional hemostatic measures required to obtain hemostasis. All device- and procedure-related AEs were collected and summarized to assess safety.

A questionnaire was completed by surgeons after their second procedure to record perceived experiences after utilizing the X1CJ and GEN11. Questions posed in relation to the X1CJ included previous experience with other advanced bipolar devices; ergonomic comfort in relation to previous device; and device usability and outcomes compared to previous device. GEN11 questions included software version; potential alarms generated; performance; and overall ease of set-up and use.

### Statistics

A summary of all performance and safety endpoints was performed for all subjects on whom the X1CJ device was utilized. The number and percentage of vessels where hemostasis was achieved (≤ Grade 3) were summarized. All device- and procedure-related AEs reported were coded to the Medical Dictionary for Regulatory Activities (MedDRA) and summarized.

## Results

A total of 40 subjects were enrolled, 20 of whom were female (50%) and all of Asian ethnicity. The patients had a mean age of 67.6 ± 11.3 years and a median BMI of 23.2 (range 18.2–30.7). Each patient had one vessel transected with the study device, for a total of 40 vessels transected. The majority of subjects were ASA class I (28/40, 70.0%), with 11 class II (27.5%), and one class III (2.5%). A lung resection was performed in all subjects for cancer, 35 (87.5%) with primary lung cancer and five with lung metastases (5/40, 12.5%). No subject received neoadjuvant chemotherapy. The mean procedure time was 2.5 ± 0.7 h with 62.5% of cases performed thoracoscopically (25/40), none of which required conversion to open thoracotomy. Mean EBL was 39.5 mL (range 0–515 mL), and no patients required blood transfusion intra- or post-operatively. Vessel skeletonization was performed in 80% of transections; inflamed or calcified vessels/tissues were observed in 5.0%; adhesions reported in 5.0%; and zero patients had atherosclerotic or fibrotic tissues.

Twenty-seven subjects had their vessel prophylactically clipped prior to transection with the study device, per surgeon preference as their reported standard practice. Vessel diameter was in the range < 3 mm (15/40, 37.5%), 3–5 mm (21/40, 52.5%), and > 5–7 mm (4/40, 10.0%). Transections included pulmonary artery branches in 70.0% (28/40), pulmonary vein branches in 20.0% (8/40), and bronchial arteries in10.0% (4/40). Hemostasis was achieved in 97.5% of vessel transections (39/40). Thirty-seven of the vessel sealings resulted in a hemostatic Grade 1 (92.5%). Two vessel sealings resulted in hemostasis Grade 3 (5%) and required minor touchup with monopolar electrosurgery. Neither of the Grade 3 vessels had prophylactic clips placed, and both subjects had a total EBL less than 35 mL. The remaining vessel (a pulmonary artery branch, 3–5 mm) did not have a hemostatic seal (Grade 4, 1/40, 2.5%), but had been prophylactically clipped and did not result in significant blood loss (the patient had a total of 5 mL EBL for the entire procedure). This vessel was further treated with a suture ligature. A full description of all vessel transections is depicted in Table 1.

**Table 1:** Vessel transection information

Variable	Category/statistic	*N* = 40 cases
Total number of vessels transected	Yes	40
Hemostasis grading scale	Grade 1	37 (92.5%)
	Grade 2	0 (0.0%)
	Grade 3	2 (5.0%)
	Grade 4	1 (2.5%)
Hemostasis achieved (Grade 3 or lower)	Yes	39 (97.5%)
	95% CI of proportion	86.8%,99.9%
Surgeon determination of diameter size range	< 3 mm	15 (37.5%)
	3–5 mm	21 (52.5%)
	> 5–7 mm	4 (10.0%)
Was an image captured with the vessel in an open jaw of the X1CJ device perpendicular to the vessel?	Yes	40 (100.0%)
Vessel transected	Pulmonary arteries branches	28 (70.0%)
	Pulmonary veins branches	8 (20.0%)
	External jugular vein	0 (0.0%)
	Other	4 (10.0%)
Grade 3 vessels using compression^a^	Yes	1 (50.0%)
Number of Grade 3 vessels using monopolar touch-up^a^	Yes	2 (100.0%)
Grade 3 vessels using monopolar touch-ups^a^	Mean (SD)	1.0 (0.0)
	Median (min, max)	1.0 (1.0, 1.0)
Number of Grade 3 vessels using bipolar touch-ups^a^	Yes	0 (0.0%)
Number of Grade 3 vessels using X1CJ touch-up^a^	Yes	0 (0.0%)
Number of vessels transected as Grade 4^b^	Yes	1 (100.0%)
Name of vessel transected as Grade 4^b^	Pulmonary artery branches	1 (100.0%)
Number of additional hemostatic measure used to achieve hemostasis^b^	Mean (SD)	1.0
	Median (min, max)	1.0 (1.0, 1.0)
Type of additional hemostatic measure used to achieve hemostasis^b^		
Sutures	Yes	1 (100.0%)

^a^Denominator is number of Grade 3 vessel transections

^b^Denominator is number of Grade 4 vessel transections

In addition to the study of primary vessel sealing, the X1CJ was also used for cutting or dissection in 65.0% (26/40) of the procedures (see Table 2). Other specific tasks performed using the study device included tissue cutting (57.5%, 23/40), tissue grasping (17.5%, 7/40), division of lymphatic tissue bundles (57.5%, 23/40), adhesiolysis (5.0%, 2/40), and tissue dissection (57.5%, 23/40). All surgeons reported satisfaction/neutral in terms of tissue grasping (100.0%). The majority of surgeons expressed satisfaction/neutral with tissue cutting (95.7%). Likewise, most were satisfied/neutral with the ability of X1CJ to divide lymphatic bundles (95.7%). A full listing of surgeon ratings of per-task usability scores is shown in Table 3.

**Table 2:** Intra-operative variables

Variable	Category	*N* = 40 cases
Occurrence of vessel skeletonization?	Yes	32 (80.0%)
Was there prophylactic use of clips or sutures as standard of surgical care before vessel transection?	Yes	27 (67.5%)
Presence of inflamed tissue or calcified tissues/vessels	Yes	2 (5.0%)
Presence of calcified tissues/vessels	Yes	0 (0.0%)
Presence of atherosclerotic tissue	Yes	0 (0.0%)
Presence of fibrotic tissue	Yes	0 (0.0%)
Presence of adhesions	Yes	2 (5.0%)
Was an Ethicon trocar used with X1CJ (if applicable)?	Yes	19 (47.5%)
Was X1CJ used for tissue cutting or dissection	Yes	26 (65.0%)
Surgical approach	Open	15 (37.5%)
	Laparoscopic	25 (62.5%)
Conversion to open if laparoscopic case	No	25 (100.0%)
Procedure duration (h)	Mean (SD)	2.5 (0.7)
	Median (min, max)	2.5 (1.5, 4.1)
	Number (missing)	40 (0)
Volume of estimated intra-operative blood loss (mL)	Mean (SD)	39.5 (90.5)
	Median (min, max)	9.5 (0.0, 515.0)
	Number (missing)	40 (0)

**Table 3:** X1CJ usage

Variable	Category	*N* = 40 cases
Were adhesions removed or divided by X1CJ?	Yes	2 (5.0%)
Surgeon’s satisfaction with the adhesion removal or division by X1CJ?^a^	Satisfied/neutral	1 (50.0%)
Were lymphatics bundles divided by X1CJ?	Yes	23 (57.5%)
Surgeon's satisfaction with the lymphatics bundles divide by X1CJ?^a^	Satisfied/neutral	22 (95.7%)
Were tissue bundles divided by X1CJ?	Yes	26 (65.0%)
Surgeon's satisfaction with the tissue bundle divide by X1CJ?^a^	Satisfied/neutral	25 (96.19%)
Was the X1CJ used for tissue grasping?	Yes	7 (17.5%)
Surgeon's satisfaction with the tissue grasping by X1CJ?^a^	Satisfied/neutral	7 (100.0%)
Was the X1CJ used for tissue cutting?	Yes	23 (57.5%)
Surgeon's satisfaction with the tissue cutting by X1CJ?^a^	Satisfied/neutral	22 (95.7%)
Was the X1CJ used for tissue dissection?	Yes	23 (57.5%)
	No	17 (42.5%)
Surgeon’s satisfaction with the dissection by X1CJ?^a^	Satisfied/neutral	21 (91.2%)
Was there use of any other energy device (monopolar, traditional bipolar, advanced bipolar, ultrasonic) during the primary procedure?	Yes	34 (85.0%)
Type of any other energy device utilized^b^	Monopolar	34 (100.0%)
	Traditional bipolar	0 (0.0%)

^a^Denominator represents the corresponding usage of X1CJ

^b^Denominator represents the corresponding usage of any other energy device (monopolar, traditional bipolar, advanced bipolar, ultrasonic) during the primary procedure

One serious adverse event deemed possibly device related was reported (2.5%) during the course of the study, a chylothorax which required an extension of the subject’s hospital stay. The subject was placed on a low-fat diet, treated with intrathoracic administration of picibanil (a sclerosing agent and immunostimulant), and was subsequently discharged. Zero patients experienced post-operative bleeding. There were no deaths during the study period.

All surgeon respondents reported they had previously utilized another advanced bipolar device, the Ligasure Maryland, with one surgeon indicating previous use of the Ligasure Blunt Tip device. Surgeons provided an assessment of the usability of the study device after their 2nd procedure (*n* = 9 surgeon questionnaires completed). When compared with the previous advanced bipolar device utilized, surgeons reported they were satisfied/neutral with the X1CJ for:

1. Less hand fatigue 55.5%
2. Reduced need for instrument changes 88.9%
3. Easier to use 55.5%
4. Performed better 55.5%
5. Cut and seal button easily distinguishable 44.4%

In addition, clinicians reported that there were zero specific GEN11-related alarms generated during any procedure. The majority of surgeon’s indicated that the GEN11 performed as intended (agree/neutral 97.5%) and stated the touchscreen allowed for easy set-up and use (agree/neutral 100.0%).

## Discussion

The use of advanced bipolar devices in thoracic surgery has been increasing in recent years, due to a relatively short device learning curve and ability to simplify minimally invasive segmentectomy [2]. Research has shown that advanced energy devices can provide burst pressures sufficient to withstand physiological tension along with less intra-operative blood loss, surgeon stress, and drainage volume [10]. The findings of adequate burst pressure, lowered blood loss and drainage have been found in a separate study of an advanced bipolar device [11]. The older version of the device, the X1 Large Jaw Tissue Sealer, was studied in a variety of open procedures, including thoracic, and was found to provide a high rate of hemostasis and few adverse events [12].

The ENSEAL X1 Curved Jaw Tissue Sealer advanced bipolar device received FDA approval in 2020, and has improved on its predicate device with uniform compression and electrical impedance feedback [13]. The present study demonstrates efficacy of the X1CJ in performing thoracic blood vessel transection in a prospective case series. Over 97% of blood vessels sealed with the device required minimal to no additional hemostatic intervention for successful transection, similar to the results observed for the X1 Large Jaw device in open thoracic procedures (96.7%) [9]. Only three vessel seals required any intervention at all, and importantly, those subjects each had less than 35 mL of total blood loss during the procedure and no transfusions were required. This amount of EBL is well within the range of other published studies on thoracic advanced bipolar device use [10, 11, 14]. One SAE, a chylothorax, occurred during the course of the study. The incidence of post-thoracic surgery chylothorax is reported to be between 0.5–1.0% [15, 16]. Thus, the rate of chylothorax of 2.5% observed in our study is near the range of other published studies [17, 18]. There is literature to suggest that the use of vessel sealing devices in thoracoscopic procedures may lower the incidence of chylothorax [17].

The low rate of product-related AE’s in this thoracoscopic case series is similar to the rate observed with the X1 Large Jaw device in open thoracic procedures (0%) [9]. Of further interest is literature which suggests that the utilization of vessel sealing devices in thoracic procedures not only may improve hemostasis but also could reduce surgeon stress, intra-operative bleeding, post-operative drainage, and drainage duration when compared to the conventional endostapler [10, 17, 19]. Further, a prospective randomized trial performed by Bertolaccini found that while no differences were observed in drain removal day and length of stay, when compared to a stapler, a vessel sealing device actually conserved functional lung tissue by allowing for superior customization of lung resections [20].

Besides demonstrating efficacy, our study also suggests surgeon satisfaction with the device performance in various tasks including division of lymphatics, division of tissue bundles, and tissue grasping, cutting and dissection. These results are consistent with previously published preclinical usability testing [13], and clinical testing of the ergonomically identical X1 Large Jaw device [9]. The majority of surgeons also reported that the X1CJ was as good or better than other ABP devices they had previously used in a variety of domains including reduced hand fatigue, reducing instrument exchanges, ease of use, and performance of critical tasks.

## Conclusion

The ENSEAL X1 Curved Jaw Tissue Sealer in combination with the GEN11 Generator demonstrated safe and effective performance in this post-market case series of thoracic vessel sealing with no reports of significant intra-operative or post-operative hemorrhage.
